# Arousal-based pupil modulation is dictated by luminance

**DOI:** 10.1038/s41598-022-05280-1

**Published:** 2022-01-26

**Authors:** Jasmine Pan, Michaela Klímová, Joseph T. McGuire, Sam Ling

**Affiliations:** 1 Psychological and Brain Sciences, Boston University, Boston, MA USA; 2 Center for Systems Neuroscience, Boston University, Boston, MA USA

**Keywords:** Psychology, Human behaviour

## Abstract

Pupillometry has become a standard measure for assessing arousal state. However, environmental factors such as luminance, a primary dictator of pupillary responses, often vary across studies. To what degree does luminance interact with arousal-driven pupillary changes? Here, we parametrically assessed luminance-driven pupillary responses across a wide-range of luminances, while concurrently manipulating cognitive arousal using auditory math problems of varying difficulty. At the group-level, our results revealed that the modulatory effect of cognitive arousal on pupil size interacts multiplicatively with luminance, with the largest effects occurring at low and mid-luminances. However, at the level of individuals, there were qualitatively distinct individual differences in the modulatory effect of cognitive arousal on luminance-driven pupillary responses. Our findings suggest that pupillometry as a measure for assessing arousal requires more careful consideration: there are ranges of luminance levels that are more ideal in observing pupillary differences between arousal conditions than others.

## Introduction

Investigation of pupil size dates back to at least the Roman times^1^. Commonly considered a visual reflex, pupils are well known to respond to visual factors, such as in the case of the *pupillary light reflex*, wherein pupils constrict in response to high luminance (brightness) and dilate in response to low luminance (darkness). However, in the past century, pupillometry research has revealed that pupils also respond to *endogenous factors* that modulate arousal, including cognitive load and mental effort^2–4^—with pupils typically dilating with increased levels of arousal, load and effort. Pupillary changes with arousal have also been linked with affect, behavioral state, surprise, attention and incentives^5^.

Seminal findings supporting the notion that pupils were “windows into the mind”^6,7^ popularized pupillometry as a measure for tracking arousal and cognitive processing. Indeed, strong correlations have bridged pupil size changes with cortical and behavioral arousal states^8–11^, under isoluminant conditions, establishing pupillometry as a widely-adopted measure for indexing and tracking arousal states in both human and animal studies. Interestingly, however, the visually-reflexive aspects of pupillometry have been less considered in the vast majority of these studies, as the luminance levels employed, while typically isoluminant within studies, have often varied across studies [e.g., Nassar et al. (screen luminance at 0.457 ± 0.010 cd/m^2^); McGarrigle et al. (screen luminance at an intermediate level between 0.0019 cd/m^2^ and 123 cd/m^2^ calibrated for each observer)^12,13^]. While these studies all substantiate that pupils dilate with increased levels of arousal, little is known regarding the potential interaction between arousal-driven pupillary changes and the pupillary light reflex. For instance, it is unknown whether the modulation of pupil size by arousal differs between environmental conditions of low luminance versus high luminance.

Here, we ask whether certain luminances are, in fact, more ideal in observing pupillary changes by arousal than others. A small handful of studies have examined the interaction between luminance, and cognitive processes and emotional arousal, on pupil size^14–18^. While interactions between luminance and cognitive processes (e.g., cognitive load, short-term memory, working memory) as well as emotional arousal on pupillary responses have been reported^14–17^, there have been some conflicting results observed among these studies. For example, Peysakhovich et al. and Pfleging et al. found larger tonic, sustained, pupillary responses with cognitive load under lower luminance and illumination; meanwhile, Steinhauer et al. observed larger modulation of pupil size by sustained cognitive processing in their bright condition compared to the dark condition^14–17^. In all of these studies, however, only 2 to 3 luminance and/or illumination levels were investigated, and the 2 or 3 luminance and illumination levels chosen within a study were highly variable between studies.

Here, we aim to map the modulatory effect of arousal across a wider range of luminances, which could possibly reconcile the conflicting results observed among the previous studies. Mapping out the relationship across a range of luminances is critical in shaping our understanding of the arousal-based pupillometry literature. There have been a large variety of stimulus and environmental conditions deployed in these studies, all assuming an additive relationship between visually-determined pupillary states and arousal—a relationship that has gone largely untested.

**Figure 1:**
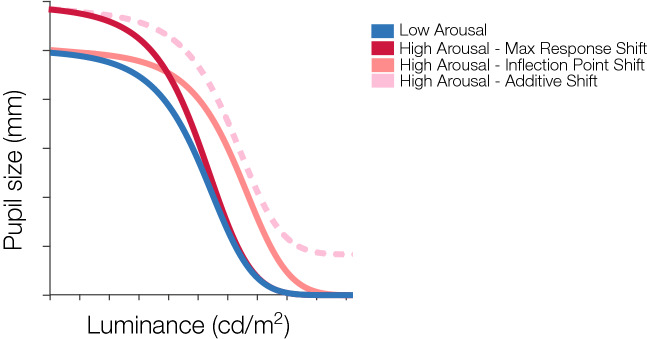
Possible effects of arousal on the pupillary light reflex response. The blue curve depicts the pupillary light response under a low arousal condition, while the different pink and red curves represent three possible modulations of the response under high arousal. The red curve illustrates a maximum response shift, in which arousal modulates pupil response at the low luminances. The solid pink curve illustrates an inflection point shift, or a horizontal shift of the curve, in which arousal’s modulation on the pupil response occurs at mid-, but not high- and low-luminances. Lastly, the dotted pink curve illustrates an additive shift, or an overall increase in pupil size with increased arousal across all luminance levels.

In this study, we sought to quantify the interaction between luminance and arousal, to determine whether this interaction is indeed additive, as has tacitly been assumed in prior work, or whether they interact multiplicatively such that arousal-driven pupillary effects are greater in some luminance regimes than in others. Pupil size is typically mapped as a function of luminance, which we refer to here as the *pupillary light function* (PLF). The human PLF has a signature nonlinear profile^19^, with the dynamic range of pupil sizes typically occurring between 2 to 8 mm. The potential modulation of the PLF by arousal can be characterized by one of a number of possible effects. Figure 1 illustrates three possible patterns of modulation of the PLF by arousal. For example, one possible effect is a consistent increase in pupil size across all luminance levels under higher levels of arousal, or an *additive shift*. Alternatively, arousal may drive multiplicative effects, such as (1) an increased pupil size primarily at lower luminances, or a *maximum response shift*, or (2) an increased pupil size only at mid-luminances, or an *inflection point shift*.

To test which of these models can best account for the relationship between the PLF and arousal, in this study we measured the pupillary light response profile under states of both high and low arousal. There are multiple forms of arousal, such as affective arousal and physical arousal, but here, we focus on *cognitive arousal*—which we define as arousal tied to cognitive factors such as effort and task difficulty. It has been suggested that affective arousal’s modulation on pupil dilation is primarily mediated by the sympathetic pathway^18^ whereas the effect of cognitive arousal on pupillary responses is predominantly mediated by inhibition on the parasympathetic system^16^, all while the pupillary light reflex is mainly mediated by a balance between the parasympathetic and sympathetic nervous system^20^. While the relationship between cognitive effort and arousal has often been examined, in which it has been argued that effort does not equate to arousal^21,22^, here we do not draw such connections. Rather, our examination of cognitive arousal is built on the idea that cognitive effort and arousal are interrelated, such that arousal is a core concept and that effort and load can drive arousal. Arousal levels as assessed through physiological measures have been shown to increase with task difficulty, oftentimes linked with increased subjective mental effort^23^.

We manipulated cognitive arousal by varying the difficulty (Hard versus Easy) of auditory arithmetic problems. By presenting problems in a continuous back-to-back stream blocked by difficulty, observers maintained a sustained state of high cognitive arousal (Hard task) or low cognitive arousal (Easy task). Studies deploying arithmetic difficulty have consistently found a modulation of pupil sizes, such that more difficult math problems evoked larger pupil size than less difficult math problems^24–28^. Such pupil dilation changes with increased difficulty implicate autonomic changes driven by the locus coeruleus-norepinephrine arousal system^29^, in which the locus coeruleus is commonly believed to play a role in the arousal system, mainly in the release of norepinephrine throughout cortex that can derive from changes in brain state (e.g., drowsiness, alertness), reward, engagement, punishment and more^9,30,31^. The pathway tied with pupillary dilation also includes the locus coeruleus, however, the link between pupillary changes with arousal within the dilation-constriction pathways is still under investigation, as there is still ongoing research mapping out the neural pathways underlying pupil dilation^5^. Moreover, whether the locus coeruleus plays a central role in the modulation of pupil size by different forms of arousal, such as cognitive arousal and affective arousal, remains an open question. Cognitive arousal’s modulation on pupillary responses could be predominantly tied to an alternative, but not exclusive, pathway to the locus coeruleus which encompasses the superior colliculus, shown to be involved in modulation of pupil size via attention-related mechanisms^32^.

In sum, we aimed to examine the potential interaction between cognitive arousal-driven pupil changes and the pupillary light reflex. To do so, we measured the PLF under high and low cognitive arousal. Our results revealed that there are indeed luminance regimes more ideal in observing pupillary differences—with high luminance being the least ideal. We also observe individual differences in the modulatory effect of cognitive arousal on the pupillary light function, with some observers showing the largest modulation at low- and/or mid-luminances and others showing equal modulation across the luminances tested. The results have implications for the selection of an ideal luminance in future experiments employing pupil size as a measure of arousal.

## Methods

### Observers

Twenty-four observers (age: 18–35; 15 females) participated in this one-session study, with the exception of one subject who completed the session over 2 days due to eye-tracker issues. Five observers were excluded from analysis due to eye-data loss, excessive eye movements and/or failure to maintain fixation. All observers provided written informed consent and had normal or corrected-to-normal vision using contact lenses. The Boston University Institutional Review Board approved the study, and the experiment was performed in accordance with relevant guidelines and regulations. Before the start of the experiment, verbal instructions and a short practice run on the task were given.

### Apparatus

The stimuli were created using MATLAB (2017b) in conjunction with the Psychophysics Toolbox^33,34^, displayed using a gamma-corrected Display +  + LCD monitor (Cambridge Research Ltd, 1440 × 1080 px, 100 Hz).

Observers sat in a room with no illumination other than the display screen and viewed the visual stimuli from a viewing distance of 116 cm with their head position stabilized with a chin rest. Observers listened to the auditory stimuli using Sennheiser HD 202 II headphones with the volume set to 50%. The left eye was recorded using an EyeLink 1000 Plus Desktop Mount (SR Research) sampled at 1000 Hz. Heart rate was recorded using Biopac’s MP160 system using a photoplethysmogram transducer attached to the observer’s index finger of choice.

### Auditory stimuli

We presented auditory math problems in order to avoid visual confounds when measuring the PLF. Auditory math problems were uniquely generated in MATLAB for each observer and produced in real-time using Psychtoolbox’s ‘Speak’ function, using the Linux operating system’s “male1” voice. In both the Easy and Hard condition, observers had to indicate whether a given math equation was true or false. The Easy condition consisted of “add 1” equations. An observer might hear, for example, “81 plus 1 equals 83”, and had to report whether the given equation was correct or incorrect. The Hard condition consisted of equations which involved subtracting a number in the 10’s digit. Here, an observer might hear, for example, “42 minus 18 equals 24”. Across the entire session, approximately 50% of the equations were true and 50% were false.

All the numbers presented in any position of the equation fell within the range of 1 to 99. For the Hard condition, the majority of the subtraction equations (78.5% across participants) involved borrowing. In order to make the Hard condition more challenging, we generated one-third of all the incorrect answers in the session to share the same last digit as the correct answer, but minus 10 or 20 the actual answer. Another one-third of the incorrect answers were ± 1, 2, or 3 the correct answer. The remaining incorrect answers consisted of a random number always less than the first number presented. In the Easy condition, one-third of all the incorrect answers in the session shared the same last digit as the correct answer, but was plus 10 or 20 the actual answer. Another one-third of the incorrect answers were plus 1, 2, or 3 the correct answer. The remaining incorrect answers consisted of a random number always greater than the first number presented.

### Procedure

**Figure 2:**
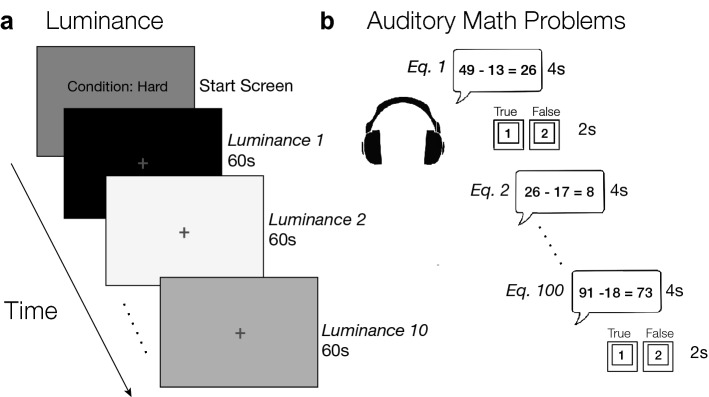
Experimental design. (**a**) Example luminance sequence for a Hard block. Participants viewed 10 different luminances presented pseudorandomly for 60 s each. (**b**) While viewing the luminances, participants simultaneously listened to and carried out solving back-to-back auditory math problems, indicating on a keyboard whether the problem given was true or false. Accuracy on the math problems were given at the end of the block.

Observers were told to maintain steady fixation at a fixation cross placed in the center of the screen (diameter: 0.1°; luminance: 15.57 cd/m^2^). In each 10-min task block, the screen cycled through 10 luminances (0.92 to 233.37 cd/m^2^, log-spaced: 0.92, 1.69, 3.13, 5.80, 10.74, 19.88. 36.81, 68.12, 126.09, 233.37), presented in a pseudorandom order for 60-s each. At the same time, observers listened to math equations (4 s), and made a button press to indicate whether the given equation was true or false (2 s). The math equation and response window (total 6 s per trial) were presented continuously throughout the block, resulting in 100 math problems per block (Fig. 2; All figures were generated and edited using MATLAB and Adobe Illustrator^35,36^). Accuracy feedback (total percent correct) was displayed at the end of each block. Observers completed a total of 4 blocks: 2 blocks of both the Hard and Easy condition, order randomized. At the end of the session, all observers completed a post-experiment questionnaire recording demographic information and perceived difficulty of the experimental conditions.

### Eye-tracking

Pupil size and gaze position were measured monocularly (left eye) using an EyeLink 1000 Plus desktop eye-tracker at a sampling rate of 1000 Hz. Pupils were measured using centroid mode and a standard 5-point calibration was run at the beginning of the experimental session.

In order to map out the pupillary light reflex response, pupil size was expressed in the absolute units of millimeters by converting EyeLink’s arbitrary units to millimeters using Hayes and Petrov’s formula^37^. Blinks were interpolated using cubic-spline interpolation^38^. Time points with abnormal pupil sizes (< 2 mm or > 9 mm) were treated as signal loss, with 1 s prior to the signal loss also excluded from analyses. For each participant, we calculated the standard deviation (SD) for both the x- and y- eye-positions to confirm fixation was being maintained. Time points where the horizontal or vertical eye-positions exceeded 2° from a participant’s x- and y-center of fixation were excluded from analysis. Four participants were excluded from further analysis due to excessive eye movements or failure to maintain fixation, with an x- and y- eye-position SD greater than 1.5° and/or having greater than 10% of data excluded from analysis. For the 19 subjects that entered analysis, the mean x- and y-center of fixation across participants were respectively − 0.4244° and − 0.1835°, and the mean percentage of data excluded from analysis was 2.89%.

For each subject and condition, mean pupil diameter in millimeters was computed for each luminance by averaging the eye traces across each 60-s luminance presentation. In terms of the pupillary light reflex, when presented with a bright luminance screen from a dark luminance screen, it takes approximately 0.2 to 1.5 s for the pupil to reach minimum constriction. When presented with a dark luminance screen from a bright luminance screen, it takes approximately 10 to 30 s for the pupil to reach maximum dilation (reviewed in^5^). When comparing different blocks of pupil sizes for a luminance level within an arousal condition, we do not see any carryover effects from the previous luminance that affects mean pupil size between the two blocks of either Hard or Easy math problems. In other words, the mean pupil size for one luminance level between the two blocks of either a Hard or Easy condition is always comparable despite the randomization of luminance presentations (Supplementary S1). We also computed the mean pupil diameter over the last 30 s of each 60-s trace in order to exclude the first half of trials where the pupil is most affected by the previous luminance level presentation and might have not yet reached steady-state pupillary size in response to the given luminance. We did not find any differences in the overall results, except for very minor pupil size differences (Supplementary S2). There is, however, a decrease in pupil size across the session observed in the majority of the observers, such that smaller pupil sizes were observed in later blocks compared to the earlier blocks of the same difficulty condition (Supplementary S1). This decrease in baseline pupil size across the progression of blocks has been observed in previous pupillometry studies, potentially arising from habituation and experience with the task, or practice effects^4,39^.

### Heart rate

Like pupil dilation, changes in heart rate have been found to accompany changes in arousal^22,29^, such that heart rate positively correlates with task difficulty^40^. This additional physiological marker was added as a secondary measure for our manipulation of cognitive arousal. During the session, pulse plethysmogram was recorded using Biopac’s TSD200 photoplethysmogram transducer, placed on the observer’s finger of choice, in conjunction with the PPG100C amplifier in the Biopac MP160 system (Biopac Systems, CA, USA). Recordings were analyzed offline using AcqKnowledge vers 5.0, where heart rate was calculated in beats per minute (BPM). Heart rate calculation in BPM was calculated using a positive peak detection, with removal of a baseline window width of 25 ms, a noise rejection of 5% of the peak and averaged over every 5 cycles. Visual artifacts were manually excluded from the BPM calculation, and all remaining abnormal heart rates (< 30 or > 150 BPM) were also excluded from further analysis conducted in MATLAB in which a time course of the BPM data were then averaged across luminance levels. Heart rate data for one observer was excluded from analysis due to data collection on two separate days. There was no significant difference in mean heart rate across the Hard and Easy conditions (Hard: 68.0648 BPM, 95% CI [62.664 73.465]; Easy: 66.5693, BPM 95% CI [56.360 76.778]; paired t-test, t(17) = 1.9699, *p* = 0.0654). Across luminance levels, such differences were only significant at three luminance levels (luminances 10.74, 36.81, 233.37 cd/m^2^; Supplementary S7).

### Analysis

**Figure 3:**
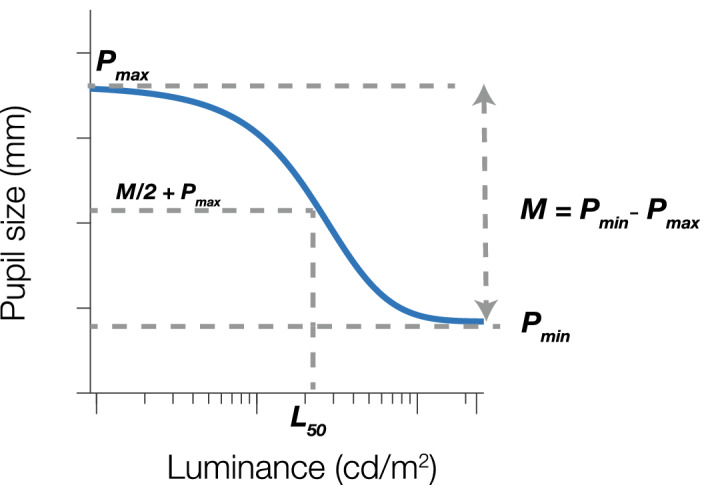
Illustration of the parameter estimates from the modified decrement Naka-Rushton function. Note that M is negative as it is a decrement curve.

The pupillary light reflex function (PLF) for each condition (Hard, Easy) exhibited a nonlinear relationship. In order to quantify the pupillary light function and its changes with arousal level to test our question of whether the relationship between luminance and arousal in influencing pupillary responses is indeed additive, as have been tacitly assumed, or whether a multiplicative interaction exists between these two factors, we fit our data with a hyperbolic ratio function, but more specifically, a modified version of the Naka–Rushton contrast response function^41–43^:where

$$ P\left( L \right) = \left( M \right)\frac{{L^{n} }}{{L^{n} + L_{50}^{n} }} + P_{max} $$

where

1 $$ M = \left( {P_{min} - P_{max} } \right) $$

where $$P\left( L \right)$$ is the pupil response as a function of luminance; $$L$$ is the luminance level; $$ P_{max}$$ is the maximum pupil response; $$L_{50}$$ is the luminance at half the maximum pupil response; and $$n $$ is the slope, or the nonlinearity in the gain of response to the input intensity. $$M$$ represents the magnitude of the PLF, which is derived by subtracting $$ P_{max}$$ from $$ P_{min}$$, or the minimum, baseline pupil response. Since the pupillary light reflex response is a decrement curve—as opposed to the Naka-Rushton function which is an increment curve—$$M$$ is negative after data fitting with Eq. (1) (Fig. 3). Taking the absolute value of $$M$$ gives the full pupillary range of the PLF curve. The pupillary light reflex curves were fit separately for both the Hard and Easy conditions using MATLAB’s *fmincon* function by optimizing the parameter estimates using nonlinear least squares, with four free parameters, $$ P_{max} , P_{min}$$, $$L_{50}$$, and $$n.$$ $$L_{50}$$ was constrained between 0.92 to 233.37 cd/m^2^. $$P_{max} $$ was constrained to the greatest mean pupil size observed across all luminances for each observer for individual fits, as $$P_{max} $$ represents the maximum pupil response in the context of our experiment, and non-saturating PLFs may lead to fits of unrealistically large $$P_{max} $$ values.

Bootstrapping analysis was used as a measure of accuracy and reliability of the parameter estimates. We sampled with replacement the data for each observer to obtain a PLF (20 data points for each luminance level) for each condition (Hard, Easy). We then computed a group-averaged PLF by averaging the data points across observers, and fitted it with the modified decrement Naka-Rushton function (Eq. 1). The parameter estimates $$ P_{max} , P_{min}$$, $$L_{50}$$, and $$n$$ were then subtracted between the Hard and Easy conditions to obtain a difference score. We repeated this 2000 times to generate a distribution of difference scores, in which p-values were then computed as a proportion of the difference scores that fell below or above zero.

## Results

**Figure 4:**
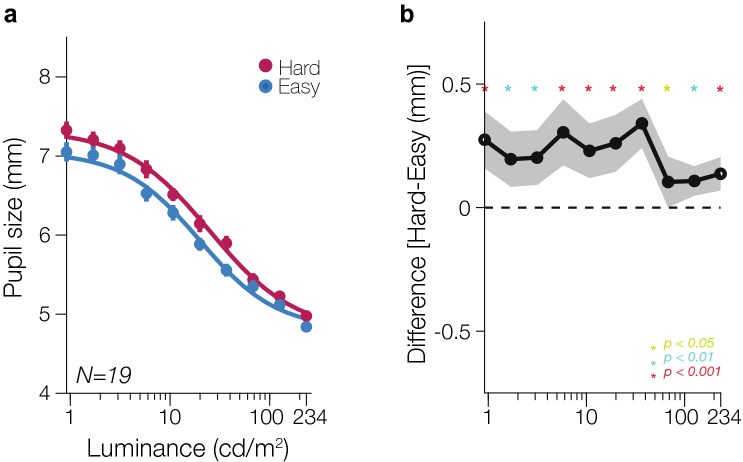
Group results. (**a**) Group-level mean pupillary light reflex function for the Hard and Easy condition fitted with the modified Naka-Rushton function. Error bars represent SEM. Overall, greater pupil size is observed in the Hard condition compared to the Easy condition across all ten luminance levels. (**b**) To test for significance between the pupil size in the Hard and Easy conditions, the difference in pupil size was taken between the Hard and Easy condition for every observer at every luminance level. Group-averaged pupil differences across all ten luminance levels were then calculated. The shaded gray area represents the 95% confidence interval.

The Hard math problems were indeed more difficult than the Easy problems (Hard accuracy: 79.71%, 95% CI [72.634 86.787]; Easy accuracy: 95.74%, CI [94.416 97.058]). Perceived difficulty (on a scale from 1–5, with 1 as easy and 5 as hard) of the Hard math problems was also higher than that of the Easy math problems (Hard perceived difficulty: 3.842, CI [3.380 4.304], Easy perceived difficulty: 1.263, CI [1.045 1.481]). We replicated previous work finding the modulation of pupil size by math problem difficulty^24–28^, wherein the Hard condition produced larger pupil diameters than the Easy condition for all individual luminance levels at the group level (Fig. 4; two-tailed t-test; *p* < 0.05) as well as larger pupil diameters in the Hard condition compared to the Easy condition when collapsed across luminance levels for all subjects (Supplementary S3; paired t-test t(18) = 9.9118, *p* < 0.001). Larger pupillary responses in the Hard condition compared to the Easy condition were also observed in group average time-courses of pupillary responses over the 6-s trials, broken down by luminance and arousal levels (Supplementary S4). However, the modulation by arousal did not appear to be uniform (Fig. 4; see also Supplementary S4, S5).

**Figure 5:**
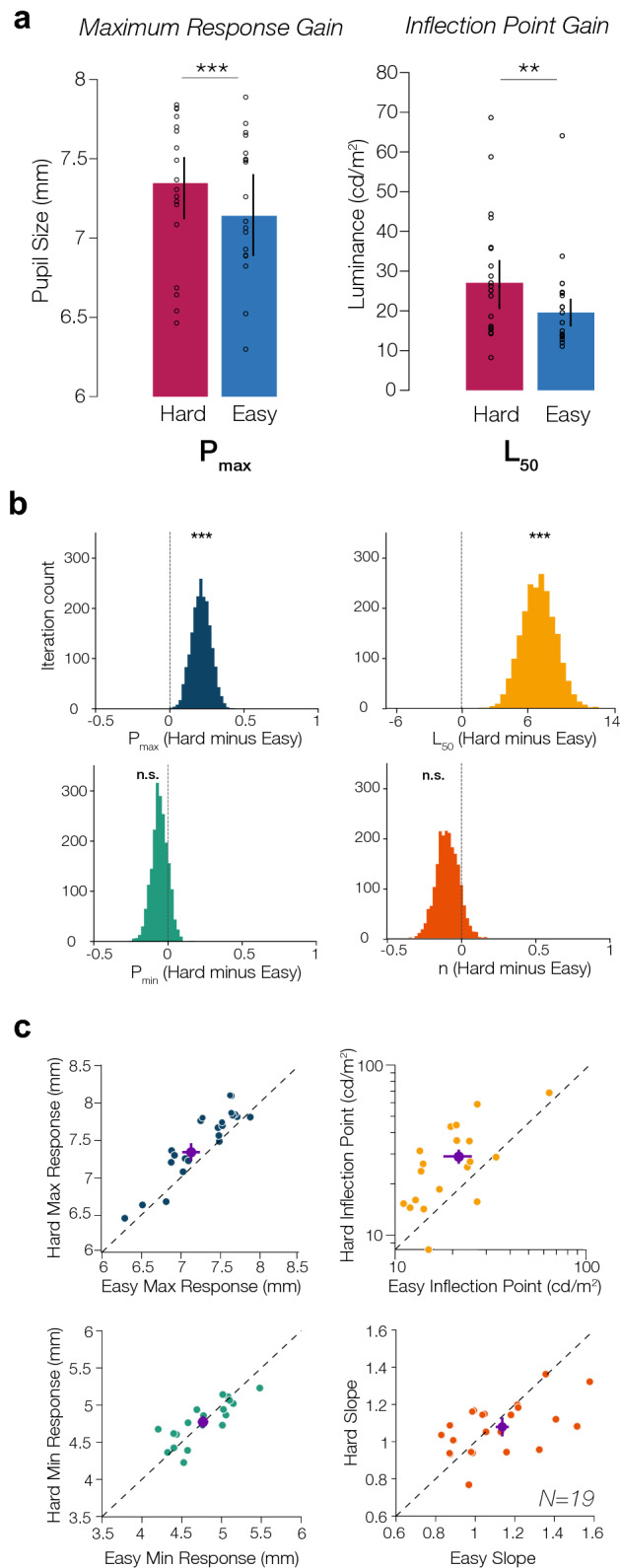
Parameter estimates. (**a**) *Left:* Mean parameter values for P_max_, maximum pupil response at lowest luminance for the Hard (red) and Easy (blue) condition. This parameter reflects a multiplicative gain of pupil size response, such that maximum pupil response is higher in the Hard condition than in the Easy condition. *Right:* Mean parameter estimates for L_50_, the inflection point or luminance value at half maximum pupil response, for both the Hard and Easy condition. Each circle represents the parameter estimate of an individual observer, and the error bar represents the 95% confidence interval. (**b**) Bootstrapped distributions (2000 iterations) of the difference between Hard and Easy parameter estimates for P_max_, L_50_, P_min_, and n. (**c**) Scatterplot of individual observer parameter estimates comparing Hard versus Easy for P_max_, maximum pupil response, L_50_, inflection point, P_min_, minimum pupil response, and n, slope. The purple dot indicates the group-averaged parameter estimates, with the error bars indicating the SEM. The dotted line indicates the unity line of no difference in parameter estimates between the Easy and Hard condition.

To quantify the PLF and its interaction with arousal, we fitted the pupillometry data with a modified decrement Naka-Rushton function (see “Methods” section). Here, the three primary modulatory effects we tested were *maximum response gain, inflection point gain,* and *additive shift*. Maximum response gain is characterized by a shift in the parameter $P_{max}$, indicating that arousal’s modulation leads to a multiplicative increase in the maximum pupil size at the lowest luminances. Inflection point gain is characterized by a shift in the parameter $L_{50}$, causing a horizontal shift of the PLF wherein arousal’s modulation on pupil response occurs solely at mid-luminances, and is absent at higher and lower luminances. Additive shift is characterized by a shift in both the $P_{max}$ and $P_{min}$ parameter, in which arousal boosts pupil size across all luminance levels. Our group results were best described as both a maximum response gain (Hard $P_{max}$ mean: 7.3495 mm, 95% CI [7.1158 7.5832]; Easy $P_{max}$ mean: 7.1393 mm, CI [6.8891 7.3896]; paired t-test, t(18) = 4.3707, *p* < 0.001), wherein arousal primarily modulated pupil size at lower luminance levels, as well as an inflection point gain (Hard $L_{50}$ mean: 26.8607 cd/m^2^, CI [20.7466 32.9748]; Easy $L_{50}$ mean: 19.469 cd/m^2^, CI [16.3717 22.5672]; paired t-test, t(18) = 2.9312, *p* = 0.0089), in which arousal primarily modulated pupil size at mid-luminance levels (Fig. 5a). Bootstrapping tests (Fig. 5b**)** for all of the parameter estimates ($P_{max}$, $L_{50}$, $P_{min} ,$ and $n$) also confirms the modulation by arousal is best characterized by a maximum response gain (bootstrap test, *p* < 0.001), as well as an inflection point gain change (bootstrap test, *p* < 0.001) on the PLF, with no difference between the Hard and Easy condition PLFs in terms of baseline pupil size (bootstrap test, *p* = 0.1700) and slope (bootstrap test, *p* = 0.0785). To obtain a clearer picture of the modulation of arousal at both the group and individual subjects’ level, Fig. 5c displays scatterplots of parameter estimates from the model-fits for each observer between the Hard and Easy condition. We found a reliable increase in $P_{max}$, or maximum pupil response, in the Hard condition across majority of the observers. Similarly, we observed a sizeable increase in $L_{50}$, or the luminance of inflection point, in the Hard condition for most observers. In terms of baseline pupil response, $P_{min}$, observers’ parameter estimates generally hovered around unity, indicating little to no difference in minimum pupil response as a function of arousal. We also find no difference in slope of the PLF, $n$, at a group level.

Due to variability in parameter estimates across participants, we then fit the modified Naka-Rushton to individual observer’s PLFs, assessing whether the maximum response gain, inflection point gain, additive shift gain, or a combination of the different models (4 possible combination of models) best characterized the modulation of arousal within individuals. To test this, the data were fit to additional modified versions of the modified decrement Naka-Rushton function where we add an additional arousal coefficient, *A*^44,45^ to test for arousal’s modulation of the PLF.

The maximum response gain model (A_R_) equation was expressed as:

2 $$ P\left( L \right) = \left( {P_{min} - [P_{max} *A]} \right)\frac{{L^{n} }}{{L^{n} + L_{50}^{n} }} + [P_{max} *A] $$

where the additional arousal parameter *A* modulates the gain of the $P_{max}$ parameter, leading to a multiplicative effect or vertical shift of the curve on the overall response (see red curve in Fig. 1).

The inflection point gain model (A_IP_) equation was expressed as:

3 $$ P\left( L \right) = \left( {P_{min} - P_{max} } \right)\frac{{(A*L^{n} )}}{{(A*L^{n} ) + L_{50}^{n} }} + P_{max} $$

where the additional arousal coefficient *A* scales multiplicatively with luminance levels $L^{n}$, leading to a horizontal shift of the curve (see solid pink curve in Fig. 1).

The additive gain model (A_A_) equation was expressed as:

4 $$ P\left( L \right) = \left( {P_{min} - P_{max} } \right)\frac{{L^{n} }}{{L^{n} + L_{50}^{n} }} + [P_{max} *A] $$

where the additional arousal parameter *A* scales $P_{max}$, leading to an overall shift of the curve across all luminances (see dotted pink curve in Fig. 1). In addition to these three core models, we fit four additional models which consisted of all the possible combinations of the three models listed above.

In order to assess which model best captured individual observer’s arousal modulation on the PLF, the Easy (low arousal) data were first fit with the modified decrement Naka–Rushton equation (Eq. 1). The parameter estimates obtained ($P_{max}$, $L_{50}$, $P_{min} ,$ and $n$) were then treated as fixed parameters when fitting the Hard (high arousal) data with the 7 models described above (e.g., Eqs. 2–4 and combination models). When fitting the Hard data with each of the 7 models, the additional arousal parameter, $A,$ was the only parameter we obtained the best estimates for. To evaluate the most parsimonious model to account for the modulatory effect of arousal on the data, we then calculated the corrected Akaike Information Criterion (AIC_c_), using sum of square errors (SSE). The corrected AIC was used as it better takes into account smaller sample sizes (N < 30)^46^. In the selection of the best parsimonious model, we then calculated ∆AIC_c_, by subtracting the minimum AIC_c_ value out of all seven models from all the other models’ AIC_c_ values. The closer a ∆AIC_c_ value is to zero, the better the model is believed to explain the data, compared to other models.

**Figure 6:**
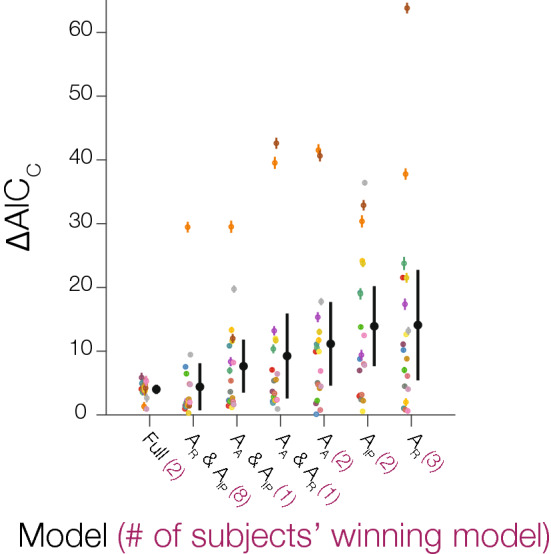
Bootstrapped model comparisons. Each black dot displays the mean ∆AIC_c_ across observers with the error bar representing the 95% confidence interval. Each colored dot represents an individual subject’s ∆AIC_c_ and the error bar represents the 95% confidence interval after bootstrapping. Model abbreviations: A_R_ is maximum response gain, A_IP_ is inflection point gain, and A_A_ is additive shift modulation. The purple number next to the model is the number of subjects’ winning model.

For every observer, we resampled with replacement the PLF for each condition (Hard and Easy) for 2000 iterations, and conducted model comparisons on each iteration to obtain confidence intervals around each model estimate. Model comparisons revealed different winning models across groups of observers: 8 observers had the maximum response gain and inflection point gain combination model as the most parsimonious model, 3 observers had the maximum response gain model, 2 observers had the inflection point model, 2 observers had the additive gain model, 2 observers had the full model, 1 observer had the additive and inflection point gain combination, and 1 observer had the maximum response gain and additive combination model. The winning model for each observer well-characterized the modulation by arousal on individual subjects’ PLFs (group-averaged R^2^ = 0.97309, CI [0.9649 0.9813]). Overall, the majority of the subjects’ modulation is best described as a combination of both a maximum response gain and inflection point gain—consistent with our group results. However, there is also a diversity of winning models across the remaining observers, which indicates different patterns of modulations across observers. Some observers show a modulation by arousal across all luminances (additive), others only at low luminances (maximum response gain) or at mid-luminances (inflection point gain), and others show a mixture of modulatory effects (see Supplementary S6 for examples of different winning modulations on individual PLFs). However, despite the heterogeneity in winning models across observers, the ∆AIC_c_ test revealed that the full model is overall the best model in explaining the data across observers (Full model ∆AIC_c_: 4.010, 95% CI [3.301 4.719]; Maximum response & Inflection point ∆AIC_c_: 4.404, CI [0.728 8.080]; Additive & Inflection point ∆AIC_c_: 7.6377, CI [3.487 11.789]; Additive & Maximum response ∆AIC_c_: 9.2419 [2.593 15.891]; Additive ∆AIC_c_: 11.1430, CI [4.601 17.686]; Inflection point ∆AIC_c_: 13.9058, CI [7.638 20.174]; Maximum response ∆AIC_c_: 14.0908, CI [5.433 22.749]; Fig. 6), with the maximum response gain and inflection point gain combination model coming closely in second.

## Discussion

In this study, we examined the potential interaction between cognitive arousal and luminance on pupillary responses. In our experiment, cognitive arousal was manipulated using auditory math problems of two difficulty levels, Hard and Easy, while observers viewed a display cycling through various luminance levels. Doing so allowed us to capture the full pupillary light response function under two different arousal levels without any visual confounds in the mapping of the pupillary light response. Quantification of the pupillary response function and its changes with cognitive arousal level revealed that cognitive arousal’s modulation on pupil size multiplicatively interacted with luminance—with the greatest modulation overall occurring at low- and mid-luminances, and little to no modulation occurring at high-luminances. However, on an individual subject’s level, there was heterogeneity in arousal’s modulation across participants, with some participants showing the greatest modulation at low-luminances, mid-luminances, equal modulation across all luminances, or different combinations of the modulations listed. Despite individual variability, across all participants, mid-luminances are always modulated by cognitive arousal.

Our finding that the greatest modulation by cognitive arousal on pupil size occurs across low and mid-luminances is consistent with previous studies that examined the interaction between luminance and cognitive effects, such as cognitive load^15,17^. These studies also observe larger sustained pupil size differences at lower luminances when compared to a higher luminance condition, demonstrating that the pupillary light response is dominant over the cognitive modulation of the pupillary response at higher luminances. However, why this occurs remains an open question, and future studies can aim towards investigating the mechanism and function underlying why generally smaller (constricted) pupil sizes when viewing high luminances are tied to smaller pupil modulation by arousal and cognitive factors.

In our current study, we focused on examining overall absolute pupil size, containing both *tonic*—absolute baseline pupil size indexing general arousal state—and *phasic*—transient and task-evoked, often indexing cognitive or emotional processing expressed in pupil size change from baseline—pupillary responses as an index of general sustained cognitive arousal state. Based on our results, we suggest that mid-luminances (~ 5 to 37 cd/m^2^) may be most ideal for pupillometry studies examining the effect of cognitive arousal on pupil size. On a group level, the greatest modulation was found at both low- and mid-luminances; however, on an individual level, there was variability in the modulation by cognitive arousal. A majority of the observers displayed a modulation by arousal at low luminances; however, there were a few observers that displayed no modulation at low luminances. Nonetheless, consistent across all observers was a modulation at mid-luminances, as observers who displayed the greatest modulatory effect at low luminances also showed some effect at mid-luminances.

Our findings are best applied to experiments that want to include pupillometry as a physiological measure of cognitive arousal between conditions by looking at overall baseline and/or absolute pupil size differences. Here, we examined the effect of sustained cognitive arousal on pupillary light responses using arithmetic problems of varying difficulty levels. Whether a similar modulation is observed using different tasks or forms of arousal when examining its interactions with luminance on pupil size is currently unknown. Furthermore, whether our results translate to phasic, task-evoked, pupillary responses also remains an open question. Peysakhovich et al. 2017 examined separately the effects of tonic and phasic pupil responses to luminance and cognitive arousal, using an n-back task as well as a math task. They observed an impact of luminance in the arousal modulation for tonic pupillary responses, but not for phasic pupil responses under the two luminance conditions they tested (~ 11 vs. ~ 28 cd/m^2^). However, the authors suggest that the lack of an effect may be due to the two luminance levels they chose, both of which are mid-luminance levels. Future studies can test whether different tasks and types of arousal (e.g., affective arousal) show the same or a different type of modulation on the PLF and on task-evoked pupillary responses over a wide-range of luminances.

Additionally, future extensions of the work could test a wider range and more extreme levels of luminances, as the luminances tested in the current study ranged between 0.92 and 233.37 cd/m^2^. Nonetheless, we do not believe testing a wider range of luminances would affect our results. A smaller modulation on pupil size was already found at the higher luminances (> 100 cd/m^2^) in our experiment, and thus we expect either a smaller or no modulation of arousal to occur at even higher luminances. However, it would be interesting to test the small subset of observers in our experiment who show the pattern of an overall additive increase in pupil size across all luminances at even higher luminances, and observe what pattern of results observers who already show little to no modulation at the higher luminances would show at even higher luminances. Future studies could also examine the modulation of cognitive arousal on the pupillary light reflex (pupillary response to light or higher luminance) versus the pupillary dark reflex (pupillary response to dark or lower luminance), across a range of different luminances. Indeed, previous work has found that cognitive load, using a pro-saccade and an anti-saccade task, modulates the pupillary dark reflex, but not the pupillary light reflex, in distractor processing^47^. In our current study, due to the randomization of our luminance presentations, we were unable to examine whether there are differences in modulation of the pupillary dark versus light reflex with our cognitive arousal manipulation.

The field of pupillometry has been gaining traction within the past decade, and employing pupillometry as a physiological measure of arousal is now widely-adopted. Studies that employed pupillometry have all carefully controlled for luminance, a potential confound in pupillary responses, within the context of the experiment; however, are there certain luminance levels that are more ideal in observing pupillary differences between arousal conditions than others? In the present study, we find that across participants the largest modulatory effect by cognitive arousal was observed at low- and mid-luminances and the smallest effect was observed at higher luminances; meanwhile, there is variability between individuals whether the modulation by cognitive arousal was observed at low luminances. Taken together, high luminances is the least ideal for studies that want to use pupillometry as a measure of cognitive arousal, and mid-luminances (~ 5 to 37 cd/m^2^) may be most ideal, as mid-luminances are always modulated by cognitive arousal across all participants.

Nonetheless, qualitatively distinct individual differences in the modulatory effect of cognitive arousal on the pupillary light function suggest that selection of an *ideal luminance* for pupillometry studies manipulating arousal is more complicated than previously thought, as there is no single luminance level that works best across all participants. We suggest that when selecting a luminance level, or comparing results across different studies—or even between individuals—considering individual differences during the formation of an experiment and interpretation of the results is important. Our experiment is the first to observe the interaction between arousal and luminance across a wide-range of luminances, and carries implications for the design and interpretation of studies employing pupillometry. Future work can aim to examine different forms of arousal (e.g., affective arousal) and its interaction with luminance on pupil response, and disentangle the mechanisms underlying such interactions and the different modulatory effects on the pupil.

## Supplementary Information

Supplementary Information.

## Data Availability

Data are available from the corresponding author upon request.
