# Supplementary material for: Arousal-based pupil modulation is dictated by luminance
Source: Sci Rep. 2022 Jan 26;12:1390. doi: 10.1038/s41598-022-05280-1 (PMC8792027; doi:10.1038/s41598-022-05280-1)
Supplement: Supplementary file 1 — Supplementary Information. [file 41598_2022_5280_MOESM1_ESM.pdf]

Supplementary Information

Arousal-based pupil modulation is dictated by luminance

Jasmine Pan, Michaela Klímová, Joseph T. McGuire & Sam Ling

a

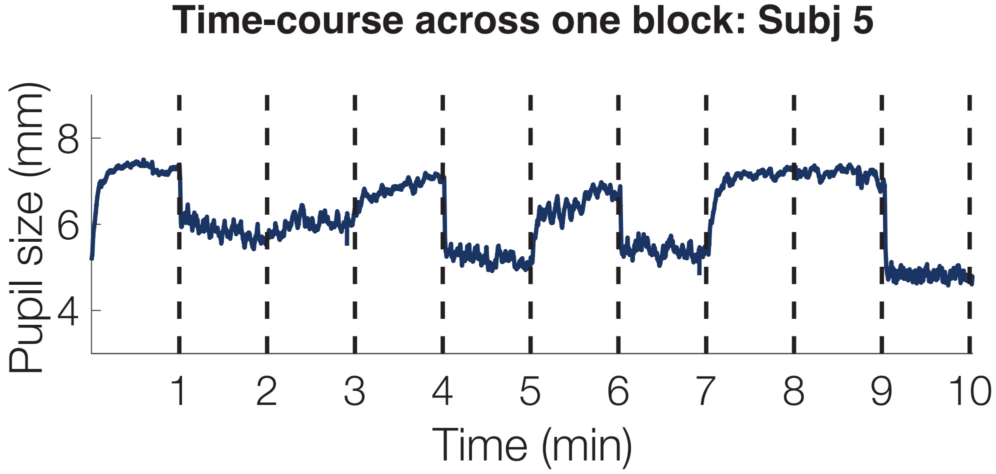

b

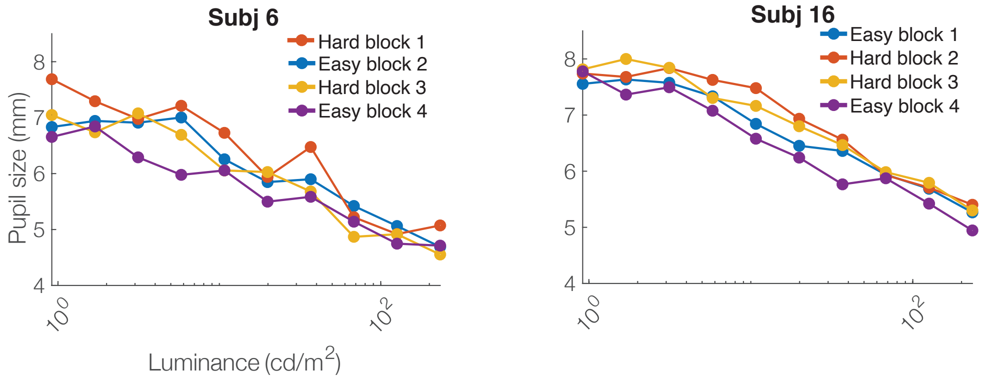

S1. Pupil size across session. (A) Pupil traces across one block for one example subject. The dashed line represents when a new luminance level was presented. The luminance level was presented pseudorandomly within each block. (B) Mean pupil size across luminance levels split up by block order for two example subjects. Later blocks (yellow and purple) showed a decrease in pupil size compared to earlier blocks (blue and red), with the difference being apparent when comparing the blocks of the same difficulty. That is, comparing the blue and purple curves for the Easy condition, and red and yellow curves for the Hard condition. Additionally, regardless of the order of difficulty of the blocks, and luminance levels order presented within blocks, mean pupil size calculated across the 60 second luminance presentation between the two blocks of any given condition are comparable, with the exception of decreased pupil size in the later blocks.

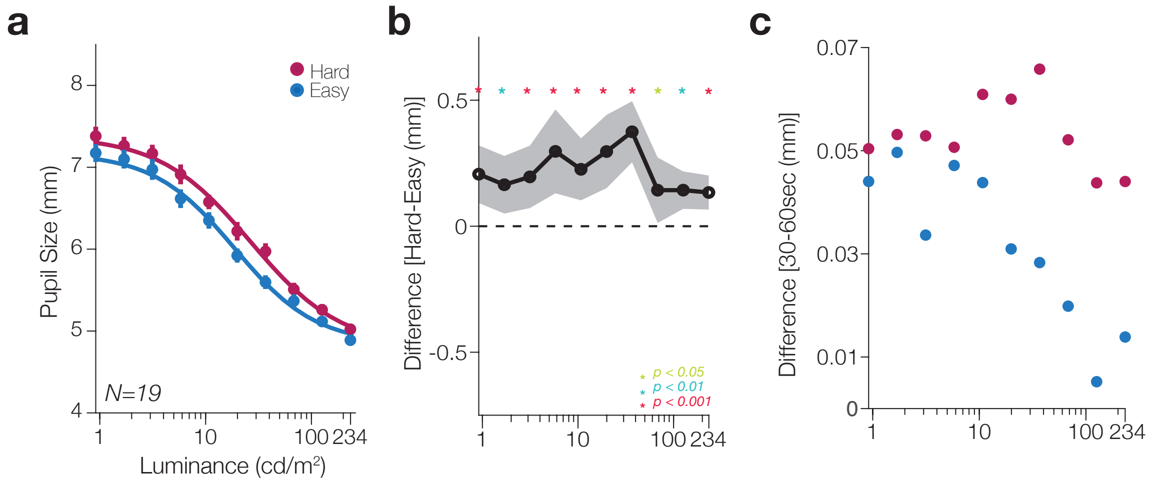

S2. Group results of analysis conducted on the last 30-seconds of a luminance. (A) Group-level mean pupillary light reflex function for the Hard and Easy condition fitted with the modified Naka-Rushton function. Error bars represent SEM. (B) To test for significant differences in pupil size between the Hard and Easy conditions, the difference in pupil size was taken between the Hard and Easy condition for every observer at every luminance level. Group-averaged pupil differences across all the luminance levels were then calculated. The shaded gray area represents the 95% confidence interval. (C) The difference in pupil size between the 60-sec versus 30-sec analysis at each luminance level for both the Hard and Easy condition. The difference was taken by subtracting the pupil size obtained from the 60-sec analyses from the pupil size obtained from the 30-sec analyses. Compared to the group results conducted over the entire 60-seconds of a luminance (Fig 4; Group Results), there is a minimal difference in the overall results with only minor pupil size differences.

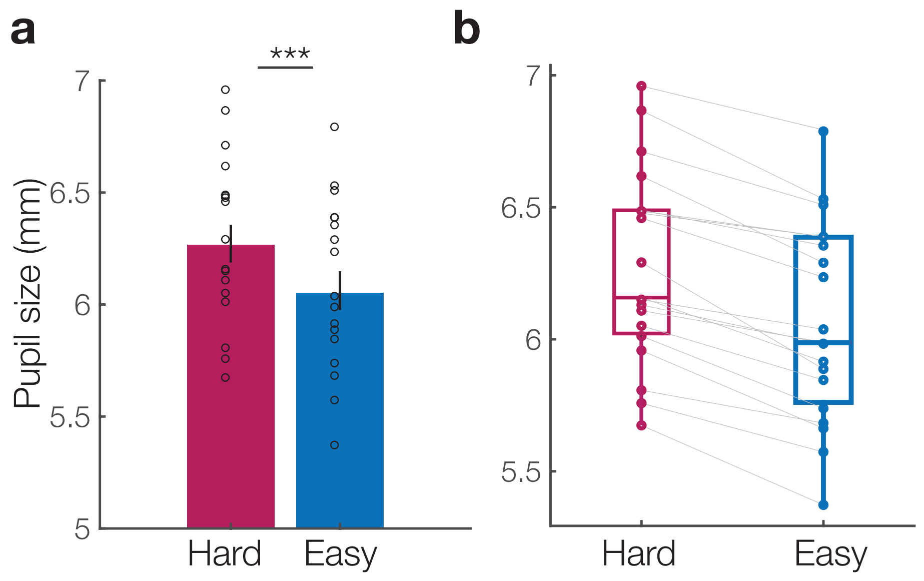

S3. Pupil Size Differences Between the Hard and Easy condition. (A) Mean pupil size in the Hard vs. Easy condition, collapsed across luminances. (B) Boxplot of mean pupil size between the Hard and Easy condition, collapsing across luminances. Overall, there is an increase in pupil size in the Hard condition when compared to the Easy condition, consistent across all participants.

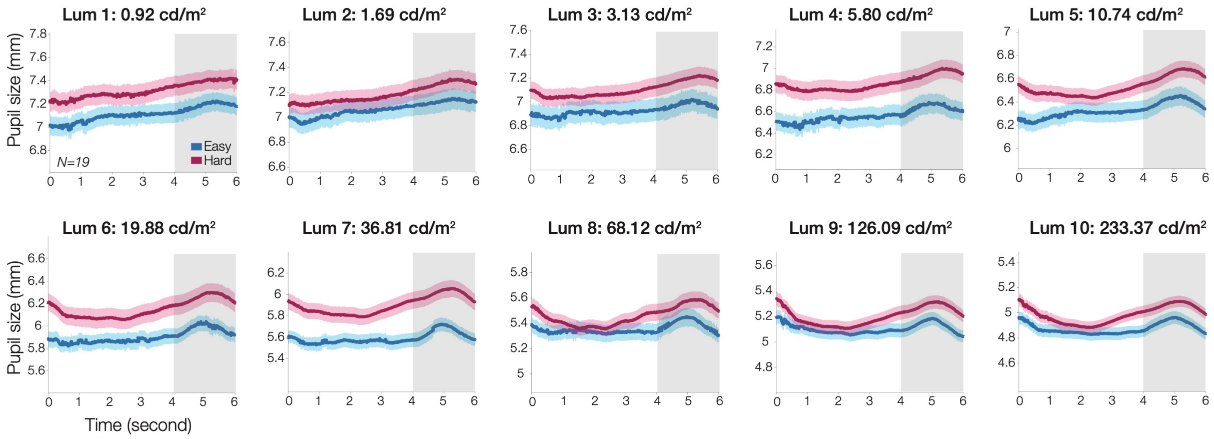

S4. Group average pupillary time-courses. Mean pupil time-courses across the 6-sec trial, which includes one math equation and the response period, broken down by luminance and arousal condition. The red (Hard) and blue (Easy) shaded area represents SEM. Pupil size in the Hard condition is higher than the Easy condition across the entire 6-sec regardless of luminance. However, certain luminances show larger modulation of pupil size by cognitive arousal than others, which is also reflected in the group pupillary light reflex functions (Fig 4; Group Results). Across most luminances, the peak pupil response occurs within the 4-6 second timeframe (light gray shaded area). This timeframe comprises the end of the auditory math equation presentation and the response period.

Luminance	0.92	1.70	3.14	5.80	10.74	19.89	36.81	68.12	126.09	233.38
Difference Mean (mm)	0.2554	0.1797	0.1763	0.3114	0.2086	0.2673	0.3523	0.1107	0.1046	0.1309
Upper 95% CI	0.3697	0.2925	0.2788	0.4517	0.3155	0.3880	0.4537	0.2181	0.1672	0.2015
Lower 95% CI	0.1412	0.0669	0.0739	0.1710	0.1017	0.1465	0.2508	0.0032	0.0420	0.0603

S5. Confidence intervals for mean pupil differences between conditions. Chart of difference values in mean pupil size between the Hard and Easy condition at every luminance level and the 95% confidence interval values.

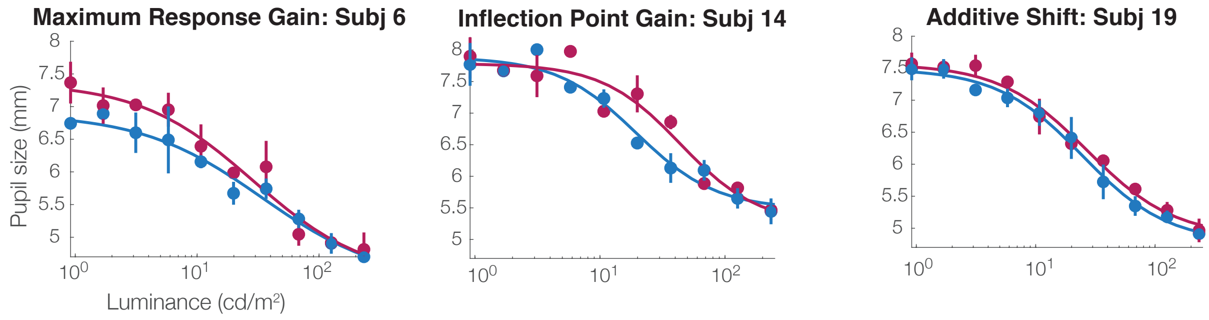

S6. Example subjects with different patterns of arousal modulatory effect on the PLF. Three example subjects each with different models that best describe the modulation of arousal on the PLF.

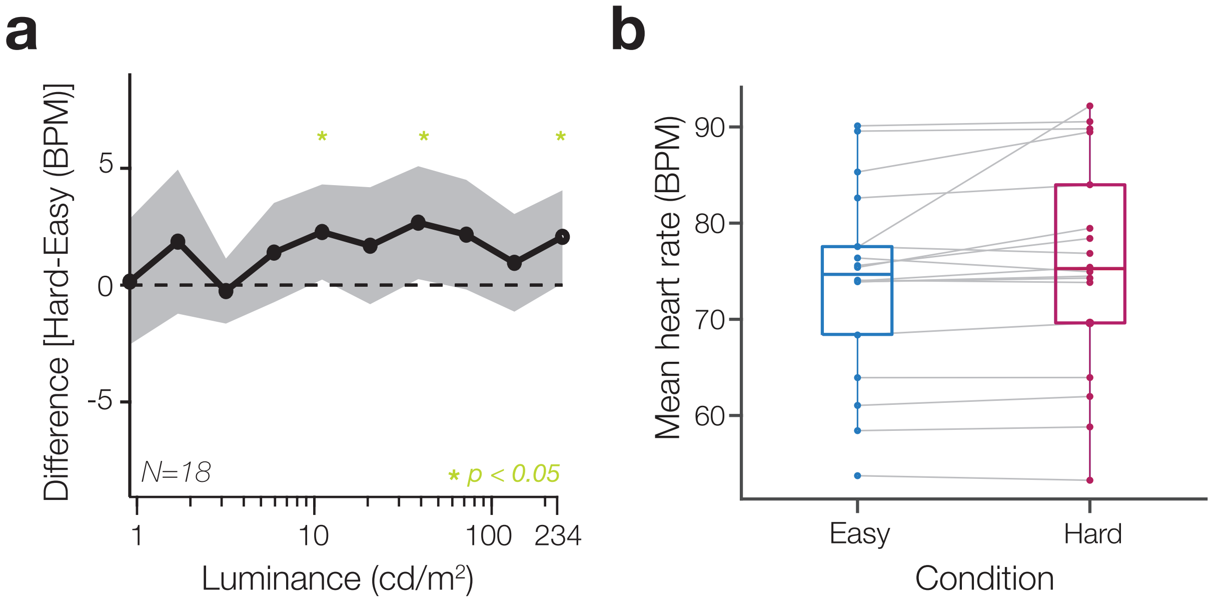

S7. Heart Rate (A) Group-average heart rate (BPM) difference between the Hard and Easy condition across the all the luminance levels. The shaded gray area represents the 95% confidence interval. The green asterisk represents significant difference between Hard and Easy condition BPM, $p<0.05$. (B) Boxplot of mean heart rate between the Hard and Easy condition, collapsing across luminances. Overall, there is a slight increase in heart rate in the Hard condition when compared to the Easy condition. However, there is individual variability in heart rate differences, with some observers showing little to no difference between conditions and others showing an increase in heart rate in the Hard condition.

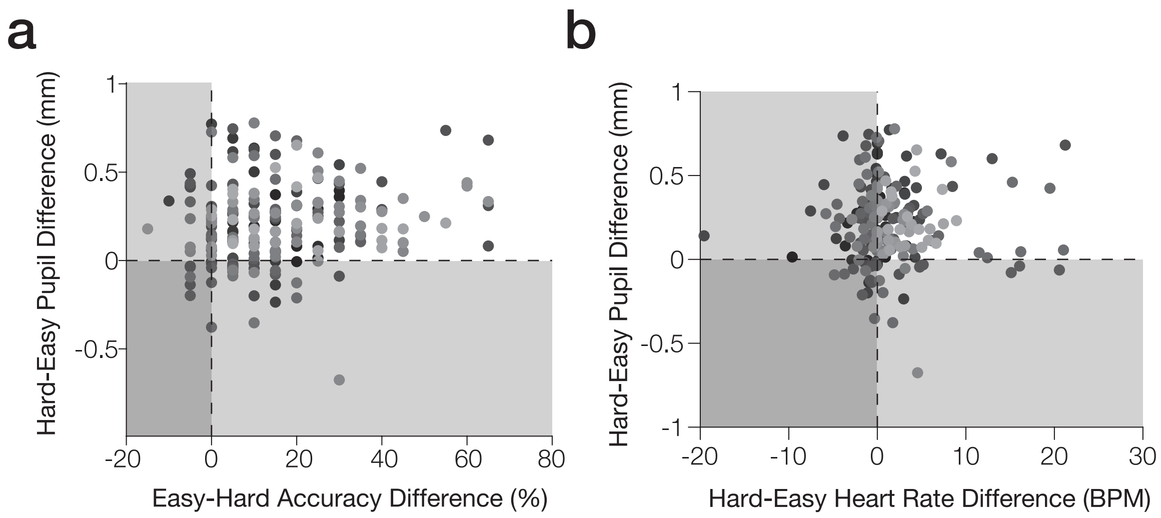

S8. Scatterplot examining the relationship between the *difference in pupil size* between the Hard and Easy condition at every luminance level for every observer as a function of (A) the *difference in accuracy* and (B) *difference in heart rate* between the Hard and Easy condition at every luminance level for every observer. There is no correlation between pupil size differences and accuracy as well as heart rate (BPM) differences.
